# Supplementary material for: Role of CaMKIIa reticular neurons of caudal medulla in control of posture
Source: Commun Biol. 2025 Oct 14;8:1469. doi: 10.1038/s42003-025-08967-z (PMC12521512; doi:10.1038/s42003-025-08967-z)
Supplement: Supplementary file 2 — Description of Additional Supplementary Files [file 42003_2025_8967_MOESM2_ESM.pdf]

Description of Additional Supplementary Files

File name- Supplementary Data 1

File description - Source data for all figures.

File name- Supplementary Video 1

File description – Mouse standing on a horizontal platform before and after unilateral activation of CaMKIIa-RNs.

File name- Supplementary Video 2

File description - Mouse standing on a horizontal platform before and after unilateral inactivation of CaMKIIa-RNs.

File name- Supplementary Video 3

File description - Mouse standing on a tilting platform before and after unilateral activation of CaMKIIa-RNs.

File name- Supplementary Video 4

File description - Mouse standing on a tilting platform before and after unilateral inactivation of CaMKIIa-RNs.
