# Supplementary material for: Role of CaMKIIa reticular neurons of caudal medulla in control of posture
Source: Commun Biol. 2025 Oct 14;8:1469. doi: 10.1038/s42003-025-08967-z (PMC12521512; doi:10.1038/s42003-025-08967-z)
Supplement: Supplementary file 8 — Reporting Summary [file 42003_2025_8967_MOESM8_ESM.pdf]

nature portfolio

Corresponding author(s): Li-Ju Hsu

Last updated by author(s): Sep 9, 2025

Reporting Summary

Nature Portfolio wishes to improve the reproducibility of the work that we publish. This form provides structure for consistency and transparency in reporting. For further information on Nature Portfolio policies, see our Editorial Policies and the Editorial Policy Checklist.

Statistics

For all statistical analyses, confirm that the following items are present in the figure legend, table legend, main text, or Methods section.

n/a	Confirmed	
☐	☒	The exact sample size (*n*) for each experimental group/condition, given as a discrete number and unit of measurement
☐	☒	A statement on whether measurements were taken from distinct samples or whether the same sample was measured repeatedly
☐	☒	The statistical test(s) used AND whether they are one- or two-sided<br>*Only common tests should be described solely by name; describe more complex techniques in the Methods section.*
☐	☒	A description of all covariates tested
☒	☐	A description of any assumptions or corrections, such as tests of normality and adjustment for multiple comparisons
☐	☒	A full description of the statistical parameters including central tendency (e.g. means) or other basic estimates (e.g. regression coefficient) AND variation (e.g. standard deviation) or associated estimates of uncertainty (e.g. confidence intervals)
☐	☒	For null hypothesis testing, the test statistic (e.g. *F*, *t*, *r*) with confidence intervals, effect sizes, degrees of freedom and *P* value noted<br>*Give P values as exact values whenever suitable.*
☒	☐	For Bayesian analysis, information on the choice of priors and Markov chain Monte Carlo settings
☒	☐	For hierarchical and complex designs, identification of the appropriate level for tests and full reporting of outcomes
☒	☐	Estimates of effect sizes (e.g. Cohen's *d*, Pearson's *r*), indicating how they were calculated

Our web collection on statistics for biologists contains articles on many of the points above.

Software and code

Policy information about availability of computer code

Data collection	ZEN 3.5, Simi Motion 9.1.1. No custom code was developed for data acquisition.
Data analysis	Software used: MATLAB R2017a, Excel 2016, ImageJ 1.54f, ZEN 3.5, CorelDRAW 2020, Shotcut 22.12.21, Simi Motion 9.1.1.

For manuscripts utilizing custom algorithms or software that are central to the research but not yet described in published literature, software must be made available to editors and reviewers. We strongly encourage code deposition in a community repository (e.g. GitHub). See the Nature Portfolio guidelines for submitting code & software for further information.

Data

Policy information about availability of data

All manuscripts must include a data availability statement. This statement should provide the following information, where applicable:

- Accession codes, unique identifiers, or web links for publicly available datasets
- A description of any restrictions on data availability
- For clinical datasets or third party data, please ensure that the statement adheres to our policy

The datasets generated during and/or analyzed during the current study are available from the corresponding author upon reasonable request.

Research involving human participants, their data, or biological material

Policy information about studies with human participants or human data. See also policy information about sex, gender (identity/presentation), and sexual orientation and race, ethnicity and racism.

Reporting on sex and gender	Not applicable.
Reporting on race, ethnicity, or other socially relevant groupings	Not applicable.
Population characteristics	Not applicable.
Recruitment	Not applicable.
Ethics oversight	Not applicable.

Note that full information on the approval of the study protocol must also be provided in the manuscript.

Field-specific reporting

Please select the one below that is the best fit for your research. If you are not sure, read the appropriate sections before making your selection.

☒ Life sciences ☐ Behavioural & social sciences ☐ Ecological, evolutionary & environmental sciences

For a reference copy of the document with all sections, see nature.com/documents/nr-reporting-summary-flat.pdf

Life sciences study design

All studies must disclose on these points even when the disclosure is negative.

Sample size	We did not perform a formal power analysis to determine sample size. Instead, sample sizes were based on prior studies using similar experimental approaches (e.g., Cregg et al., 2020, Nature Neuroscience; Bouvier et al., 2015, Cell). The robustness and magnitude of the observed effects suggest that the sample sizes used were sufficient to detect meaningful differences.
Data exclusions	No data were excluded from the analysis.
Replication	All experimental results were replicated in multiple runs of experiments with consistent results.
Randomization	Animals were assigned to experimental groups based on cage distribution rather than formal randomization. However, sex was balanced across groups where applicable.
Blinding	Blinding was not performed. Experimenters were aware of group allocation during both data collection and analysis. This was due to clear and distinguishable behavioral phenotypes (e.g., trunk roll tilt) that made blinding infeasible.

Reporting for specific materials, systems and methods

We require information from authors about some types of materials, experimental systems and methods used in many studies. Here, indicate whether each material, system or method listed is relevant to your study. If you are not sure if a list item applies to your research, read the appropriate section before selecting a response.

Materials & experimental systems

n/a	Involved in the study	
☐	☒	Antibodies
☐	☐	Eukaryotic cell lines
☐	☐	Palaeontology and archaeology
☐	☒	Animals and other organisms
☐	☐	Clinical data
☐	☐	Dual use research of concern
☐	☐	Plants

Methods

n/a	Involved in the study	
☐	☐	ChIP-seq
☐	☐	Flow cytometry
☐	☐	MRI-based neuroimaging

Antibodies

Antibodies used	Rabbit anti-RFP (Rockland, Cat# 600-901-379, RRID: AB_2209751); Chicken anti-RFP (Rockland, Cat# 600-401-379, RRID:

Antibodies used	AB_10704808); Rabbit anti-DsRed (Clontech, Cat# 632496); Alexa Fluor 568-conjugated anti-rabbit IgG (Jackson ImmunoResearch, Cat# 111-585-003)
Validation	The antibodies used have been validated in numerous publications and with Western/IHC on mouse samples as indicated on manufacturer's product page.

Eukaryotic cell lines

Policy information about cell lines and Sex and Gender in Research

Cell line source(s)	Not applicable.
Authentication	Not applicable.
Mycoplasma contamination	Not applicable.
Commonly misidentified lines (See ICLAC register)	Not applicable.

Palaeontology and Archaeology

Specimen provenance	Not applicable.
Specimen deposition	Not applicable.
Dating methods	Not applicable.

☐ Tick this box to confirm that the raw and calibrated dates are available in the paper or in Supplementary Information.

Ethics oversight	Not applicable.

Note that full information on the approval of the study protocol must also be provided in the manuscript.

Animals and other research organisms

Policy information about studies involving animals; ARRIVE guidelines recommended for reporting animal research, and Sex and Gender in Research

Laboratory animals	The following mouse strains were used in this study: wild-type C57BL/6J, GAD67-Cre, and Vglut2-Cre (Jackson Stock 016963), and . All mice were of both sexes and aged 8–12 weeks at the time of experiments. Animals were housed in standard cages (4–5 per cage) under a 12/12 hour light/dark cycle at 23–24 °C and 45–65% humidity, with food and water available ad libitum.
Wild animals	No wild animal was used
Reporting on sex	Both male and female mice were used in this study. Sex was not a variable in study design, and no sex-specific analyses were performed. Sex was determined by visual inspection of external genitalia prior to experiments.
Field-collected samples	No field-collected samples were used
Ethics oversight	All animal experiments and procedures were approved by the local ethical committee, Stockholm's Norra Försöksdjursnämnd. These experiments were performed in accordance with European guidelines for the care and use of laboratory animals.

Note that full information on the approval of the study protocol must also be provided in the manuscript.

Clinical data

Policy information about clinical studies

All manuscripts should comply with the ICMJE guidelines for publication of clinical research and a completed CONSORT checklist must be included with all submissions.

Clinical trial registration	Not applicable.
Study protocol	Not applicable.
Data collection	Not applicable.
Outcomes	Not applicable.

Dual use research of concern

Policy information about dual use research of concern

Hazards

Could the accidental, deliberate or reckless misuse of agents or technologies generated in the work, or the application of information presented in the manuscript, pose a threat to:

No	Yes	
☒	☐	Public health
☒	☐	National security
☒	☐	Crops and/or livestock
☒	☐	Ecosystems
☒	☐	Any other significant area

Experiments of concern

Does the work involve any of these experiments of concern:

No	Yes	
☒	☐	Demonstrate how to render a vaccine ineffective
☒	☐	Confer resistance to therapeutically useful antibiotics or antiviral agents
☒	☐	Enhance the virulence of a pathogen or render a nonpathogen virulent
☒	☐	Increase transmissibility of a pathogen
☒	☐	Alter the host range of a pathogen
☒	☐	Enable evasion of diagnostic/detection modalities
☒	☐	Enable the weaponization of a biological agent or toxin
☒	☐	Any other potentially harmful combination of experiments and agents

Plants

Seed stocks	Not applicable.
Novel plant genotypes	Not applicable.
Authentication	Not applicable.

ChIP-seq

Data deposition

☐ Confirm that both raw and final processed data have been deposited in a public database such as GEO.

☐ Confirm that you have deposited or provided access to graph files (e.g. BED files) for the called peaks.

Data access links<br>*May remain private before publication.*	Not applicable.
Files in database submission	Not applicable.
Genome browser session<br>(e.g. UCSC)	Not applicable.

Methodology

Replicates	Not applicable.

Sequencing depth	Not applicable.
Antibodies	Not applicable.
Peak calling parameters	Not applicable.
Data quality	Not applicable.
Software	Not applicable.

Flow Cytometry

Plots

Confirm that:

- [ ] The axis labels state the marker and fluorochrome used (e.g. CD4-FITC).
- [ ] The axis scales are clearly visible. Include numbers along axes only for bottom left plot of group (a 'group' is an analysis of identical markers).
- [ ] All plots are contour plots with outliers or pseudocolor plots.
- [ ] A numerical value for number of cells or percentage (with statistics) is provided.

Methodology

Sample preparation	Not applicable.
Instrument	Not applicable.
Software	Not applicable.
Cell population abundance	Not applicable.
Gating strategy	Not applicable.

- [ ] Tick this box to confirm that a figure exemplifying the gating strategy is provided in the Supplementary Information.

Magnetic resonance imaging

Experimental design

Design type	Not applicable.
Design specifications	Not applicable.
Behavioral performance measures	Not applicable.

Acquisition

Imaging type(s)	Not applicable.
Field strength	Not applicable.
Sequence & imaging parameters	Not applicable.
Area of acquisition	Not applicable.

Diffusion MRI [ ] Used [ ] Not used

Preprocessing

Preprocessing software	Not applicable.
Normalization	Not applicable.
Normalization template	Not applicable.

Noise and artifact removal | Not applicable.

Volume censoring | Not applicable.

Statistical modeling & inference

Model type and settings | Not applicable.

Effect(s) tested | Not applicable.

Specify type of analysis: ☐ Whole brain ☐ ROI-based ☐ Both

Statistic type for inference (See Eklund et al. 2016) | Not applicable.

Correction | Not applicable.

Models & analysis

n/a	Involved in the study
☐	☐ Functional and/or effective connectivity
☐	☐ Graph analysis
☐	☐ Multivariate modeling or predictive analysis

Functional and/or effective connectivity | Not applicable.

Graph analysis | Not applicable.

Multivariate modeling and predictive analysis | Not applicable.
